# The Role of Membranes in Modern Winemaking: From Clarification to Dealcoholization

**DOI:** 10.3390/membranes15010014

**Published:** 2025-01-09

**Authors:** Carolina E. Demaman Oro, Bruna M. Saorin Puton, Luciana D. Venquiaruto, Rogério Marcos Dallago, Giordana Demaman Arend, Marcus V. Tres

**Affiliations:** 1 Department of Food and Chemical Engineering, Universidade Regional Integrada do Alto Uruguai e das Missões (URI), 1621 Sete de Setembro Av., Centro, Erechim 99709-910, RS, Brazil; 044079@aluno.uricer.edu.br (C.E.D.O.); 042316@aluno.uricer.edu.br (B.M.S.P.); venquiaruto@uricer.edu.br (L.D.V.); dallago@uricer.edu.br (R.M.D.); 2 Department of Food and Chemical Engineering, Federal University of Santa Catarina, Florianópolis 88040-900, SC, Brazil; 3 Laboratory of Agroindustrial Processes Engineering (LAPE), Federal University of Santa Maria (UFSM), 3013 Taufik Germano Rd., University II DC, Cachoeira do Sul 96503-205, RS, Brazil

**Keywords:** wine quality enhancement, microfiltration, ultrafiltration, nanofiltration, reverse osmosis, wine concentration, wine stabilization

## Abstract

The utilization of membrane technologies in winemaking has revolutionized various stages of production, offering precise and efficient alternatives to traditional methods. Membranes, characterized by their selective permeability, play a pivotal role in enhancing wine quality across multiple processes. In clarification, microfiltration and ultrafiltration membranes, such as ceramic or polymeric membranes (e.g., polyethersulfone or PVDF), effectively remove suspended solids and colloids, resulting in a clearer wine without the need for chemical agents. During stabilization, membranes such as nanofiltration and reverse osmosis membranes, often made from polyamide composite materials, enable the selective removal of proteins, polysaccharides, and microorganisms, thereby improving the wine’s stability and extending its shelf life. Additionally, in dealcoholization, membranes like reverse osmosis and pervaporation membranes, typically constructed from polydimethylsiloxane (PDMS) or other specialized polymers, facilitate the selective removal of ethanol while preserving the wine’s flavor and aroma profile, addressing the increasing consumer demand for low-alcohol and alcohol-free wines. This article provides a comprehensive analysis of the advancements and applications of membrane technologies in winemaking.

## 1. Introduction

Wine is an alcoholic beverage produced from grapes through fermentation, during which yeast converts grape sugars into ethanol. Beyond its flavor and aroma, wine is valued for its health-promoting components, including organic acids, phenols, and aromatic compounds, which exhibit antioxidant properties, support lipid metabolism, and help regulate blood sugar levels. Organic acids are particularly important in wine, enhancing its sensory profile by softening the taste, reducing astringency, and providing acidity. Additionally, they play a crucial role in stabilizing phenolic compounds, supporting antioxidant activity, and preserving the wine color. Among these, tartaric acid is the most abundant, alongside other acids such as citric, malic, succinic, lactic, and acetic acids [1].

The central role of organic acids in wine composition underscores their significance in vinification, or winemaking—the process of converting grapes into wine through fermentation. Specific techniques within vinification vary based on the type of wine and desired quality. After grape harvesting, the initial steps involve destemming and crushing the fruit. For white wines, subsequent stages include pressing, clarification, and fermentation. In red winemaking, fermentation typically occurs before or after pressing, with an optional maceration phase. Maceration is critical for red wines, as it influences their color, aroma, and flavor by extracting polyphenols from grape skins. Cold pre-fermentation maceration, which involves maintaining grapes at low temperatures (6–15 °C) for several days, is a widely used technique but requires significant energy [2].

Once fermentation is complete, red wines undergo cleaning, filtration, and maturation, often aging in oak barrels for six months to a year. This process enhances the wine by extracting compounds from the wood. In contrast, white wines are stabilized and refined in non-wood containers. Temperature changes and the presence of tartaric salts can cause wine instability, leading to tartaric precipitation. To address this, white wines may undergo cold stabilization, a process requiring low temperatures over extended periods, typically ranging from −4 °C to 0 °C for 1 to 3 weeks, which demands substantial energy. The stability of tartaric acid, or its ability to prevent tartrate precipitation, is a key factor in winemaking. Although tartrate crystals are harmless and do not alter the wine’s flavor, their appearance, resembling shards of glass, can be unappealing to consumers. Stabilization methods fall into two categories. The first, known as the “subtraction” approach, involves reducing tartrate concentrations by removing potassium (K^+^), calcium (Ca^2+^), and tartaric acid ions through technological processes. The second, the “addition” approach, uses external stabilizing agents to enhance the wine’s ability to retain tartrate and prevent precipitation [1]. For both red and white wines, subsequent stages include bottling, aging, packaging, and distribution. The length of aging and refining depends on the wine’s type and quality [2].

The complexity of wine composition further emphasizes the challenges of its production and refinement. The composition of wine is highly complex, comprising molecules of varying sizes and characteristics: (a) solute molecules (<1 nm), including ethanol, glycerol, sugars, organic acids, ions, and monomeric phenolic compounds; (b) molecules exhibiting colloidal behavior (1 nm–1 μm), such as polysaccharides, polyphenols, and other macromolecular compounds; and (c) particles (>1 μm), such as microorganisms, organic precipitates, and tartrate crystals. Furthermore, wine contains dissolved gases (e.g., O_2_ and CO_2_) and an extensive array of aromatic compounds, which significantly contribute to its sensory complexity [3].

Given the intricate composition of wine, membrane technologies have emerged as a transformative tool in modern winemaking. These technologies are redefining traditional methods by enabling cleaner, more efficient, and sustainable processes. Their ability to selectively separate components at a molecular level has proven invaluable not only in winemaking, but also across industries such as water purification, food and beverage production, pharmaceuticals, and bioengineering [4,5,6,7,8,9].

The advantage of membrane systems lies in their high selectivity. Membranes are designed to allow specific molecules or ions to pass through while rejecting others, based on parameters such as size, shape, charge, and chemical affinity. This selective permeability enables precise separation processes without chemical additives, reducing environmental impact and enhancing product purity. For instance, ultrafiltration (UF) and nanofiltration (NF) membranes can fractionate bioactive compounds or remove contaminants while preserving essential nutrients and flavors in food and beverage applications. Similarly, reverse osmosis (RO) membranes offer exceptional efficiency in desalination and water treatment, ensuring access to clean water with minimal waste generation [10,11,12,13].

Sustainability is increasingly recognized as essential within the wine industry. By adopting environmentally conscious strategies, wineries can reduce their ecological footprint and enhance their operational efficiency and business performance.

Therefore, the objective of this review is to provide a comprehensive analysis of the advancements and applications of membrane technologies in the wine sector, emphasizing their innovative contributions to improving quality, sustainability, and process efficiency. This review delves into key aspects such as wine dealcoholization and the recovery of phenolic compounds, while also presenting case studies of wineries that have successfully implemented membrane technologies. These case studies illustrate practical and measurable benefits, complemented by an evaluation of economic feasibility and environmental advantages, offering an integrated perspective on the transformative potential of these technologies in modern winemaking.

## 2. Membrane Technologies in Winemaking

**Figure 1:**
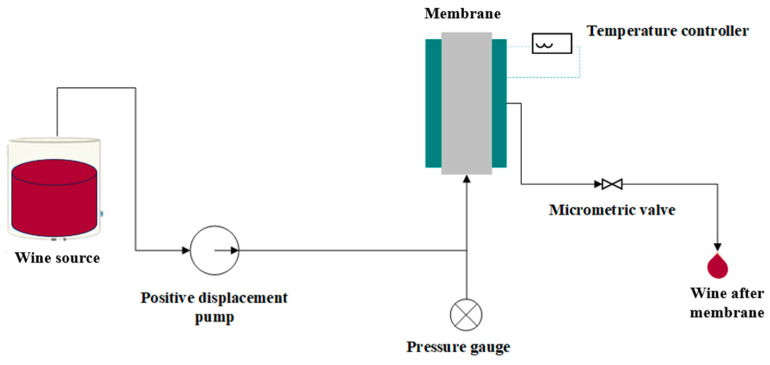
Schematic representation of the membrane filtration system.

Membrane processes are used in different stages of winemaking, including the treatment of must and wine, to promote clarification, cleaning, stabilization, sterilization, and dealcoholization. Membranes are classified according to pore size into microfiltration (MF), UF, NF, RO, and electrodialysis (ED) (Figure 1). The choice of the most appropriate process varies according to the specific stage and the desired objectives.

MF, with pore sizes ranging from 0.2 µm to 10 µm, is effective for removing suspended solids, yeast, and bacteria. UF, with smaller pore sizes between 0.01 µm and 0.1 µm, provides selective removal of proteins, polysaccharides, and fine colloids. NF, with pore sizes ranging from 0.001 µm to 0.01 µm, allows the partial removal of salts and small organic molecules while retaining flavor and aroma compounds. RO features the smallest pore sizes, typically less than 0.001 µm, and is used for dealcoholization and wine concentration by selectively removing ethanol and water. ED, although not classified by pore size, utilizes ion-selective membranes to remove ionic compounds such as potassium, calcium, and tartaric salts, with these membranes categorized based on their ionic selectivity rather than physical pore size.

Red wines predominantly feature anthocyanins and tannins, while white wines contain hydroxycinnamic acids and polysaccharides, which are derived from grapes, yeasts, and fungi. Proteins in wine originate from grapes and yeast autolysis, with red wines having minimal protein content due to precipitation by tannins. In contrast, white and rosé wines may contain proteins in concentrations ranging from 10 to 500 mg/L. Filtration is a critical step in winemaking, aimed at reducing the turbidity caused by suspended macromolecules and particles, improving microbiological stability. Traditional methods such as sheet and diatomaceous earth filtration are widely used, but cross-flow MF is gaining popularity due to its ability to combine clarification, stabilization, and sterile filtration in a single process while eliminating diatomaceous earth usage [12,14,15,16,17].

MF membranes, typically with pore sizes of 0.2 µm, are used for red wines, achieving microbiological stability and clarity (turbidity < 1 NTU) while preserving sensory qualities. For white wines, MF membranes with pore sizes between 0.1 and 0.22 µm ensure lower turbidity (<0.5 NTU) and more stable filtration fluxes due to their ability to exclude larger particles. Among various polymeric MF membranes, hydrophilic cellulose acetate (0.2 and 0.45 µm) has demonstrated superior performance by reducing the adsorption of polyphenols and polysaccharides compared to polyethersulfone (PES) or polypropylene membranes [3].

Despite its advantages, cross-flow MF is hindered by fouling, which reduces permeation flux and impacts process economics and wine quality. Fouling is influenced by wine composition, operational conditions, and interactions between wine colloids and the membrane. Polysaccharides, for instance, contribute significantly to fouling, not due to their total content but their structure and composition. Highly branched polysaccharides, such as arabinogalactans, and those of high molecular weight or with specific side-chain arrangements tend to adsorb more readily onto membrane surfaces. This is attributed to increased molecular interactions and steric hindrance, which exacerbate fouling by creating dense, gel-like layers that reduce permeation flux.

Membrane fouling during MF processes typically results in a significant decline in flux. Initial flux values for cross-flow MF in winemaking generally range from 50 to 150 L/m^2^·h under optimal conditions. Still, depending on wine composition and operational parameters, they can drop to 20 to 50 L/m^2^·h after prolonged fouling.

Protein precipitation in membrane processes, particularly UF, is driven by a combination of size exclusion and physicochemical interactions between proteins and the membrane surface. Proteins with hydrophobic regions can adsorb onto hydrophobic membrane surfaces, contributing significantly to fouling and reducing filtration efficiency. Additionally, particles such as yeast and fines exacerbate fouling by forming adherent cakes that block the membrane pores, further hindering performance.

Membrane material is another key factor influencing fouling and flux behavior. Polypropylene membranes show higher flux and lower adsorption of polysaccharides and polyphenols than polyethersulfone membranes, likely due to their lower surface energy and reduced hydrophobic interactions. Hydrophilic materials, such as cellulose acetate, also perform better in mitigating fouling because they reduce the adhesion of hydrophobic compounds and colloids. The chemical structure of the membrane material significantly impacts fouling mechanisms, with smoother surfaces and hydrophilic chemistries generally associated with lower fouling rates and more stable flux over time.

UF is a versatile option for wine clarification, employing membranes with molecular-weight cut-offs (MWCOs) between 5 and 10 kDa. These membranes effectively remove macromolecules such as phenolics and proteins, which are associated with astringency, while maintaining critical wine parameters such as pH, sulfur dioxide levels, and viscosity. However, UF can sometimes lead to over-clarification, removing colloidal matter that is vital for preserving flavor intensity and overall wine quality. The performance of UF is influenced by size exclusion and other interactions between wine components and the membrane surface. These interactions include hydrophobic and electrostatic forces, as well as hydrogen bonding, which can result in membrane fouling and impact the selectivity of the filtration process. Phenolic compounds, for instance, may adsorb onto the membrane due to hydrophobic interactions, while polysaccharides can form gels on the surface, exacerbating fouling. Additionally, interactions between the membrane material and sulfur dioxide or other stabilizing agents in wine may alter the filtration efficiency and longevity of the membrane [18,19,20,21,22].

Recent advances in membrane technology have sought to address these challenges. Systems like the Oenoflow XL-A, which utilizes poly(vinylidene fluoride) (PVDF) hollow-fiber membranes, and Flavy FX, which employs hydrophilic polyethersulfone (PES) membranes, are designed with backflushing capabilities to mitigate fouling. These materials are engineered to resist polyphenol and polysaccharide adsorption, ensuring greater durability and performance. Research also highlights the advantages of hydrophilic membranes with asymmetrical structures, demonstrating reduced fouling tendencies and the better preservation of wine’s sensory characteristics.

Continued innovation at the laboratory scale focuses on optimizing membrane materials and cleaning protocols to further enhance the efficiency of UF for wine clarification. These developments hold promise for balancing effective macromolecule removal with the retention of compounds critical for flavor and quality, making UF a continually evolving and essential technology in modern winemaking [23].

## 3. Dealcoholization

### Ethanol Removal Process in Wine

The demand for low-alcohol and alcohol-free wines has been experiencing significant growth globally, driven by changing consumer preferences towards healthier lifestyles and increased awareness of the health risks associated with excessive alcohol consumption. The global non-alcoholic wine market is projected to increase from a value of USD 2.57 billion in 2024 to USD 6.94 billion by the end of 2034.

The alcohol content of wine must not fall below 8.5% vol to be classified as wine. The European Commission permits the removal of up to 2 percentage points of alcohol, provided the ethanol concentration remains at or above 8.5% vol. Products with an alcohol content lower than this threshold cannot be called “wine”. In 2021, EU Regulation No. 2117/2021 introduced two new categories for such products: “dealcoholized wine”, which must contain no more than 0.5% *v/v* ethanol, and “partially dealcoholized wine”, which has an ethanol content exceeding 0.5% *v/v* but remaining below 8.5% vol.

The alcohol levels in wine can be effectively regulated through strategies implemented throughout the winemaking process, from vineyard management to post-fermentation treatments. Post-fermentation alcohol reduction or removal relies on physical and chemical methods. Common techniques include thermal processes like vacuum distillation and spinning cone columns, membrane-based technologies such as RO, osmotic distillation (OD), and pervaporation (PV), and extraction methods using organic solvents, adsorbents, or gases. These approaches are designed to maintain the sensory integrity of the wine, ensuring that the aroma and flavor profiles remain as close to the original as possible.

Among the post-fermentation techniques, membrane technologies have revolutionized the selective removal of ethanol from wine, producing low-alcohol or alcohol-free wines while preserving the beverage’s sensory qualities. One of the most promising techniques for ethanol extraction is membrane OD. A hydrophobic membrane, typically polypropylene, separates the wine from an extracting solution, such as water [24].

OD typically operates at pressures ranging from 0.2 to 0.5 bar and temperatures between 10 °C and 25 °C, which are significantly lower than traditional distillation methods. These conditions reduce the risk of the thermal degradation of sensitive wine components. By preventing significant alterations in the concentration of minor elements such as polyphenols and organic acids, OD ensures that the wine’s flavor, aroma, and overall profile remain intact, making it a highly effective method for producing alcohol-reduced wines without compromising quality.

Moreover, OD is a promising membrane-based technique for wine dealcoholization, offering advantages such as low energy consumption and minimal impact on wine composition and sensory properties. In OD, a hydrophobic membrane (typically polypropylene) separates the wine from an extracting solution, usually water. The process relies on ethanol vapor pressure differences across the membrane, which enhance ethanol flux while retaining most wine components in the feed. OD is considered a clean technology, as the extracting solution produced contains low ethanol levels and minimal aroma compounds. However, its large water consumption—approximately 0.5 L per liter of wine—results in significant waste and economic losses, limiting its adoption in wineries. To improve OD sustainability, a separation process for the extracting solution can recycle water and concentrate ethanol into a stream suitable for use as second-generation bioethanol. This approach reduces wastewater generation and increases resource efficiency [25,26,27,28,29,30,31].

Other advanced membrane systems, such as RO and PV, have also been successfully applied to selectively remove ethanol while concentrating desirable compounds. These methods are particularly advantageous because they avoid the use of harsh chemicals or high heat, which could degrade the quality of the wine. In the study conducted by Esteras-Saz et al. [24], red wine was partially dealcoholized from 14.0% to 11.0% *v/v* ethanol using OD with pure water as the extracting agent and polypropylene membranes. The resulting extracting solution, containing 5.3% ethanol, underwent hydrophobic–hydrophilic PV to recover 88% of the ethanol and produce water with 99.4% purity. This water was successfully reused as the extracting solution in OD, achieving a dealcoholized wine with comparable aroma retention to that obtained with fresh water. The integration of OD and PV demonstrates a sustainable approach to wine dealcoholization, combining resource recovery and waste minimization.

Ethanol molecules are smaller and less polar compared to water molecules, which influences their interaction with the membrane surface. In membranes with dense structures, such as those used in RO, the small pore size restricts the passage of ethanol, resulting in higher rejection rates. This behavior is influenced by the hydrophilic or hydrophobic nature of the membrane material; hydrophilic membranes often exhibit higher water affinity, allowing water to permeate preferentially, while limiting ethanol transport.

Additionally, ethanol removal is influenced by molecular diffusion and solubility. Ethanol’s solubility in the membrane material, determined by its affinity for the polymer matrix, can impact its transport rate. For instance, membranes with higher compatibility for ethanol may allow partial diffusion, affecting overall rejection efficiency. The concentration polarization effect, where ethanol molecules accumulate near the membrane surface, also plays a role, potentially altering rejection behavior. This phenomenon emphasizes the importance of operational conditions, such as pressure and flow rate, in optimizing ethanol removal processes.

Advances in membrane technology for dealcoholization over the past 30 years have consolidated this approach as one of the most promising in the wine industry, accounting for almost 50% of the processes used [32]. This technology offers an efficient solution for reducing alcohol content, with minimal impact on the sensory and organoleptic characteristics of the wine, standing out in comparison to other methods [33]. However, several challenges remain, including maintaining wine quality at an industrial scale, optimizing membrane materials to minimize undesired interactions, and reducing operational costs. Table 1 provides an overview of studies from the literature that employed various methods for alcohol removal in wines.

Kumar et al. [37] evaluated the performance of three NF membranes (TS 40, NF99, HL) and one RO membrane (RO-SE) in the treatment of ethanol–water mixtures (0–10.5% *v*/*v*) and white wine (10.5% *v*/*v*). These studies indicated marked differences in ethanol rejection efficiency, directly related to the characteristics of the membrane material and structure. The membranes analyzed are mostly composed of polyamide in a thin film composite (TFC) structure, widely recognized for their selectivity in controlling the flow of molecules. This selectivity is determined by pore size, chemical affinity, and surface properties.

Denser membranes, such as RO-SE, have smaller pores and exhibited higher rejection rates for ethanol (10.64%), limiting their application for dealcoholization processes due to the difficulty of nonpolar molecules passing through their compact structure. Similarly, TS 40 presented high ethanol rejection (18.30%), making it equally unsuitable for this type of application. In contrast, HL and NF99 membranes, which have slightly larger pores and greater permeability, demonstrated lower ethanol rejection rates (5.46% and 5.14%, respectively). This characteristic makes them more effective in reducing the alcohol content of wine, allowing the alcohol content to be reduced to less than 1.3% *v*/*v*. In addition, these membranes favored the retention of desirable compounds, such as reducing sugars (glucose and fructose) and organic acids (citric and tartaric acid), essential for preserving the sensory quality of the wine.

The behavior of the membranes also influenced the visual and taste aspects of the concentrated wine, which presented darker hues due to the effects of concentration. The correlation between the membrane material and its efficiency in ethanol rejection reflects the fundamental role of physicochemical properties in the selective separation of components in complex mixtures, such as water–ethanol and wine, highlighting the importance of choosing the appropriate membrane for each specific application.

## 4. Recovery of Phenolic Compounds

Membrane technologies also play a crucial role in the circular economy by enabling the recovery and reuse of resources from industrial processes. The winemaking byproducts can be treated with membranes to recover valuable compounds such as bioethanol or polyphenols, turning waste into resources and promoting sustainability.

Winemaking byproducts are a valuable source of bioactive compounds, particularly polyphenols, which exhibit antioxidant and anti-inflammatory properties. These plant-derived metabolites have shown potential in preventing or treating various conditions, including neurodegenerative, cardiovascular, and kidney diseases and certain cancers. Consequently, polyphenols are highly interested in nutraceuticals, pharmaceuticals, cosmetics, and food additives [38,39,40].

Recovering polyphenols from agri-food residues involves both extraction and purification. While several extraction methods and solvents have been explored, techniques such as solid–liquid extraction, ultrasound-assisted extraction, and supercritical fluid extraction are the most feasible for industrial applications. For sectors like food, pharmaceuticals, and cosmetics, solvents must meet strict safety requirements, with water–ethanol mixtures being the most commonly used and effective [41,42,43,44,45].

Purifying polyphenols is a critical step in enhancing their value, but remains a significant challenge due to the complexity of the extracts. Membrane-based techniques, such as UF, are increasingly applied for clarification and fractionation before more selective processes like NF or RO. While UF separation ideally relies on molecular size, factors such as molecule shape, membrane material, surface interactions, and the formation of a filtration cake layer significantly influence performance [8,10,46].

Phenolic compounds contain hydroxyl groups that can engage in hydrogen bonding with hydrophilic membranes. These interactions depend on the degree of polarity of both the membrane material and the phenolic compounds. Hydrophilic membranes are particularly effective in reducing fouling caused by hydrophobic phenolics, enhancing recovery efficiency. Phenolics can carry a negative charge under specific pH conditions, influencing their rejection or passage through charged membranes. Adjusting the pH of the feed solution can optimize electrostatic repulsion or attraction, aiding in the selective recovery of desired phenolic fractions.

The efficiency of UF membranes varies depending on the nature of the extract and the system configuration. For instance, phenolic compounds from winery sludge extracts showed a 69% retention rate using a 100 kDa polysulfone membrane [47]. In comparison, total phenolic rejection in kiwifruit juice was 13.5% with a 30 kDa cellulose acetate membrane [48]. However, further research is needed to optimize membrane processes for polyphenol recovery, particularly given the complexity of such extracts.

Malolactic fermentation lees from Albariño wine production are a valuable source of polyphenols with potential applications in cosmetics, pharmaceuticals, and food products. Mir-Cerdà et al. [49] employed an environmentally friendly extraction method using water to recover phenolic acids, flavonoids, and related compounds from this winemaking byproduct. The extract was purified through MF and UF with membranes of 30 kDa and 5 kDa MWCOs. An analysis revealed that caftaric acid was the most abundant polyphenol, alongside other compounds like coutaric acids, gallic acid, and astilbin. The 30 kDa membrane preserved the extract’s composition, whereas the 5 kDa membrane reduced the polyphenolic content, making the former more suitable for processing. This sustainable approach demonstrates the potential of Albariño wine lees as a significant source of phenolic compounds, particularly phenolic acids.

## 5. Wineries Using Membrane Processes

Membrane technologies offer several advantages over traditional methods. They operate at ambient temperatures, which prevents the degradation of temperature-sensitive compounds, and they allow for the selective separation of components, preserving the wine’s essential qualities. Furthermore, these technologies are energy-efficient and environmentally friendly, as they eliminate the need for chemical additives and reduce waste.

These processes operate based on molecular mechanisms that exploit the selective permeability of membranes, which differentiate molecules based on size, charge, shape, or chemical affinity. For example, ethanol removal from wine is commonly achieved using membrane-based methods such as osmotic distillation (OD) and reverse osmosis (RO). These techniques maintain the sensory qualities of wine while reducing alcohol content.

Osmotic distillation relies on hydrophobic microporous membranes, typically made of materials like polypropylene, to facilitate the transfer of ethanol and water vapor from the wine to an extracting solution. The process is driven by a vapor pressure gradient across the membrane. At the molecular level, ethanol and water molecules evaporate at the wine–membrane interface, diffuse through the membrane as vapor, and condense into the extracting solution. The hydrophobic nature of the membrane prevents the liquid-phase transfer of wine, ensuring that non-volatile components, such as phenolics and sugars, are retained. This mechanism allows OD to preserve the wine’s flavor and aroma profile while reducing the alcohol content.

Reverse osmosis employs semi-permeable membranes, often made of polyamide, to separate ethanol and water molecules from the wine under high pressure. These membranes are designed to allow the passage of smaller molecules like ethanol and water while rejecting larger molecules such as polyphenols, organic acids, and sugars. The molecular size and diffusion dynamics are key to this selective separation. By operating at ambient temperatures, RO ensures the minimal alteration of wine’s volatile and aromatic compounds, thus maintaining its sensory characteristics.

However, proteins in wine, particularly in white and rosé wines, can cause haze and instability. Membrane filtration techniques such as microfiltration (MF) and ultrafiltration (UF) are effective in precipitating and removing these proteins, ensuring wine clarity and stability. Microfiltration employs membranes with pore sizes ranging from 0.1 µm to 0.45 µm. Proteins, being larger than the membrane pores, are retained either on the surface or within the membrane structure. At the molecular level, proteins interact with the membrane surface through hydrophobic or electrostatic forces, forming a fouling layer. While this fouling can reduce membrane performance, cleaning protocols or anti-fouling coatings are typically applied to mitigate its effects.

Fouling presents a significant challenge in the application of membrane technologies in winemaking processes, particularly during ethanol removal and protein precipitation. This phenomenon occurs when particles, macromolecules, or microorganisms accumulate on the membrane surface or within its pores, leading to decreased performance, reduced permeability, and increased operational costs. Understanding the types of fouling and implementing effective mitigation strategies are essential to maintaining the efficiency of membrane systems.

Fouling in winemaking can be categorized into four main types. Particulate fouling arises from suspended solids and colloidal particles, such as tartrate crystals and polysaccharides, that adhere to the membrane surface, forming a barrier to fluid flow. Organic fouling is caused by the accumulation of organic compounds, including polyphenols, proteins, and tannins, which interact with the membrane through hydrophobic or electrostatic forces. Biofouling occurs when microorganisms, such as yeast or bacteria, proliferate on the membrane surface, forming biofilms that obstruct filtration. Finally, scaling (inorganic fouling) is caused by the precipitation of salts, such as calcium tartrate and potassium bitartrate, which crystallize under specific pH and temperature conditions.

The impact of fouling includes reduced membrane permeability and selectivity, shorter membrane lifespans, and higher energy consumption. Additionally, fouling can compromise wine quality by introducing contaminants or altering its chemical profile. To address these challenges, several strategies can be employed, such as the following: (i) The selection of appropriate membrane materials: Using membranes with low fouling potential, such as those with hydrophilic coatings, can reduce the adhesion of organic and particulate matter. Materials resistant to biofouling, such as polyamide or modified polypropylene, are particularly effective. (ii) Pre-treatment of feed wine: Implementing clarification processes, such as sedimentation or centrifugation, helps remove suspended solids and colloids before filtration. Adjusting the pH and temperature can also minimize the precipitation of salts and organic compounds. (iii) Regular cleaning protocols: Cleaning-in-place (CIP) systems using detergents and enzymatic solutions tailored to specific fouling types are crucial. For instance, alkaline cleaners are effective against organic fouling, while acid solutions can address scaling. Alternating cleaning agents ensures comprehensive fouling management. (iv) Operational adjustments: Optimizing transmembrane pressure and flow rate minimizes particle deposition, while cross-flow filtration can reduce fouling by sweeping particles away parallel to the membrane surface. (v) Anti-fouling techniques: Periodic backflushing can help remove accumulated foulants, while chemical or UV sterilization is effective against biofouling. These techniques extend membrane lifespan and improve performance. (vi) Monitoring and early detection: The real-time monitoring of operational parameters, such as flux, pressure drop, and product quality, allows for the early detection of fouling. Regular maintenance and inspections further prevent severe fouling events [48,50,51].

However, the problem of scaling up in membrane filtration processes, especially in winemaking and other food and beverage industries, refers to the challenges faced when transitioning from laboratory or pilot-scale operations to full-scale industrial applications. While membrane technologies offer numerous benefits, such as improved product quality and energy efficiency, scaling up these processes presents several difficulties that can impact performance, cost-effectiveness, and sustainability.

As the system size increases, the accumulation of foulants (e.g., organic matter, salts, microorganisms) on the membrane surface can become more significant. Larger systems often experience higher volumes of feedstock, which can lead to increased fouling rates. In large-scale operations, fouling can quickly reduce membrane permeability, resulting in lower filtration rates, higher energy consumption, and more frequent cleaning cycles. This increases operational costs and reduces the overall efficiency of the process. Achieving uniform filtration performance across a larger system can be difficult. Variability in membrane properties, fouling rates, and operating conditions across multiple membrane modules can result in uneven filtration, which leads to inconsistent product quality. Maintaining uniform flux, product quality, and contaminant removal across all membrane units is a significant challenge in large-scale systems.

To address these challenges, several strategies can be employed. One approach is optimizing pre-treatment methods, such as sedimentation or centrifugation, to reduce fouling rates and enhance membrane efficiency. Modular system designs allow for incremental scaling, enabling capacity expansion without the need for extensive overhauls. Advanced monitoring and control systems can help maintain consistency in operation, ensuring that large-scale systems run efficiently and maintain product quality. Additionally, selecting high-performance membranes with anti-fouling properties and applying surface modifications can improve system performance and extend membrane lifespans. Finally, energy recovery systems and waste valorization strategies, such as pressure-retarded osmosis or the recycling of cleaning solutions, can help reduce energy consumption and environmental impact.

In conclusion, while scaling up membrane filtration systems in industries like winemaking offers numerous advantages, it also presents significant challenges. Addressing these challenges requires a combination of optimized process management, efficient system design, advanced membrane technologies, and sustainable energy and waste management practices. These solutions ensure that large-scale membrane filtration systems remain cost-effective, energy-efficient, and environmentally sustainable.

## 6. Conclusions

Wine production is a complex process that transforms grapes into a beverage cherished for its rich sensory attributes and health-promoting compounds, such as organic acids, phenols, and antioxidants. These components play a crucial role in defining the sensory profile of wine, enhancing stability, and delivering bioactive benefits. However, the intricate composition of wine, consisting of molecules of varying sizes and properties, presents significant challenges during production and refinement.

In response to these challenges, membrane technologies have emerged as transformative tools in modern winemaking, offering efficient, precise, and sustainable solutions. These technologies leverage selective separation principles, allowing for the filtration of molecules based on size, shape, charge, and chemical affinity. Unlike conventional methods, membrane processes do not require chemical additives, ensuring minimal environmental impact and higher product purity.

One of the most notable applications of membrane technology in winemaking is clarification. Cross-flow MF has gained popularity for its ability to simultaneously clarify, stabilize, and sterilize wine. This method effectively reduces turbidity and ensures microbiological stability without altering the sensory qualities of the wine. Membrane systems are also employed in stabilization and sterilization, removing the particles responsible for haze and instability. This enhances wine clarity and extends its shelf life.

An increasingly important application is dealcoholization, which addresses growing consumer demand for low-alcohol and alcohol-free wines. Technologies like OD enable the selective removal of ethanol while preserving the wine’s flavor and aroma profiles. These processes are energy-efficient, operate at low pressures and temperatures, and minimize the environmental footprint compared to traditional methods.

Beyond production, membrane technologies support resource recovery and sustainability, aligning with the principles of a circular economy. For instance, byproducts of winemaking, such as bioethanol and polyphenols, can be recovered using membrane systems, reducing waste and valorizing agricultural residues. Another significant advantage of membrane technologies is their energy efficiency. Compared to conventional separation processes, such as distillation, membrane systems operate under lower energy demands, reducing operational costs and environmental impact.

By integrating these advanced technologies, the winemaking industry has achieved significant improvements in product quality, sustainability, and operational efficiency. Membrane technologies not only address the technical complexities of wine production, but also meet evolving consumer preferences for sustainable and health-conscious beverages. As these innovations continue to develop, they are poised to further revolutionize winemaking and related industries, reaffirming their critical role in modern production systems.

## Figures and Tables

**Table 1:** Dealcoholization methods and initial and final alcohol contents.

Method	Type of Wine	Operating Conditions	Initial and Final Alcohol Content (% *v*/*v*)	Reference
OD	Falanghina white	Microporous hydrophobic polypropylene membrane. Feed streams flowed into the module in recycling and countercurrent mode. Wine was fed at a 70 mL/min flow rate and distilled water at 140 mL/min on the tube side. Each cycle lasted 30 min.	12.5 to 0.3	[33]
RO	Merlot red Pinot Noir rosé Chardonnay white	Alfa Laval RO98pHt M20 spiral wound composite membranes. Each wine was dealcoholized at a constant pressure of 3.5 MPa and 20 °C at a 70 mL/min flow rate	13.9 to 0.7 12.2 to 0.7 13.4 to 0.7	[34]
PV	Cabernet Sauvignon red	PDMS commercial composite membranes	12.5 to 0.5	[35]
Spinning cone column	Shiraz Sangiovese Petit Verdot Sangiovese	Main conditions: feed flow between 1793 to 3173 L/h, steam temperature 28.5 to 37.1 °C, vacuum pressure 95 kPa, and steam pressure from 17 to 432 kg/h	15.1 to 0.3	[36]

## Data Availability

No new data were created or analyzed in this study. Data sharing is not applicable to this article.
